# Efficacy and safety analysis of consolidation immunotherapy following definitive concurrent chemoradiotherapy for locally advanced esophageal squamous cell carcinoma

**DOI:** 10.3389/fimmu.2026.1983549

**Published:** 2026-10-19

**Authors:** Wanming Liu, Qian Zhu, Siyuan Xu, Juan Chen, Dianjun Wang, Ting Wu, Jun Shen, Yiyao Zhu, Zhifu Chen, Yan Liu, Guofang Wang, Chaoyang Wu, Yan Wang

**Affiliations:** 1 Department of Radiation Oncology, The People’s Hospital Affiliated to Jiangsu University, Zhenjiang, China; 2 Department of Oncology, Yangzhong People’s Hospital, Yangzhong, Jiangsu, China; 3 Danyang Hospital of Traditional Chinese Medicine, Danyang, China; 4 Department of Pathology, The People’s Hospital Affiliated to Jiangsu University, Zhenjiang, China; 5 Jurong People’s Hospital, Jurong, China; 6 College of Clinical Medicine and Medical Laboratory, Jiangsu University, Zhenjiang, China

**Keywords:** concurrent chemoradiotherapy, esophageal squamous cell carcinoma, immunotherapy, PD-L1 expression, programmed death-1 inhibitor

## Abstract

**Background:**

Definitive concurrent chemoradiotherapy (dCRT) is standard for locally advanced esophageal squamous cell carcinoma (ESCC), but recurrences remain common. Evidence for consolidation immunotherapy after dCRT is limited. This study evaluated the efficacy and safety of consolidation immunotherapy after dCRT and explored the association between programmed death-ligand 1 (PD-L1) expression and treatment outcomes.

**Methods:**

This multicenter retrospective cohort included ESCC patients who completed dCRT without progression and received consolidation chemotherapy alone or with a programmed death-1 (PD-1) inhibitor followed by maintenance (July 2019–December 2024). Inverse probability of treatment weighting (IPTW) adjusted for baseline differences. Overall survival (OS), progression-free survival (PFS), objective response rate (ORR), and treatment-related adverse events (TRAEs) were compared. PD-L1 expression was assessed by combined positive score (CPS) in evaluable tissue.

**Results:**

Among 96 patients, 45 received consolidation chemotherapy plus a PD-1 inhibitor followed by maintenance and 51 received consolidation chemotherapy alone. Median OS (43.1 vs. 26.0 months; *P* = 0.014) and PFS (25.4 vs. 9.6 months; *P* = 0.014) were longer with immunotherapy. After IPTW, OS (37.6 vs. 25.6 months; *P* = 0.018) and PFS (12.7 vs. 9.2 months; *P* = 0.037) remained significant. ORR was higher with immunotherapy (60.0% vs. 27.4%; *P* = 0.001). PD-L1 was evaluable in 38 patients; subgroup analyses were exploratory. Grade ≥3 TRAEs did not differ significantly (17.8% vs. 23.5%; *P* = 0.660).

**Conclusion:**

Consolidation chemotherapy plus a PD-1 inhibitor followed by maintenance after dCRT was associated with longer OS and PFS in locally advanced ESCC, with comparable grade ≥3 TRAEs. PD-L1 analyses were inconclusive because few patients had evaluable tissue. These findings require validation in larger cohorts.

## 1 Introduction

Esophageal cancer is one of the most common gastrointestinal malignancies worldwide (1). ESCC is the predominant histologic subtype in East Asia and accounts for more than 90% of esophageal cancer cases in China. Because early-stage esophageal cancer is often asymptomatic, many patients are diagnosed with locally advanced disease, and some are not candidates for surgical resection (1–3). For patients with unresectable, locally advanced ESCC, dCRT is the standard treatment (4). The RTOG 85-01 trial established the survival benefit of dCRT, with a 5-year OS rate of approximately 26% (5). Despite advances in radiotherapy techniques, including the transition from two-dimensional radiotherapy to intensity-modulated radiotherapy (IMRT), local recurrence and distant metastasis remain the main patterns of treatment failure after dCRT (6). The 2-year OS rate remains only 38%–44.3% (1). Therefore, effective consolidation strategies are urgently needed to improve long-term survival in this patient population.

Immune checkpoint inhibitors (ICIs) have improved clinical outcomes in first-line and second-line treatment for advanced esophageal cancer, supporting their evaluation in earlier disease stages (7–9). Radiotherapy can induce immunogenic cell death (10) and modulate the tumor microenvironment, thereby enhancing antitumor immune responses and potentially synergizing with ICIs (11–14). This potential synergy provides a biological rationale for using ICIs as consolidation therapy after dCRT. The PACIFIC trial demonstrated a survival benefit with consolidation durvalumab after concurrent chemoradiotherapy in patients with unresectable stage III non-small cell lung cancer (15). This treatment paradigm has prompted the investigation of consolidation immunotherapy after dCRT in patients with locally advanced ESCC (16).

However, current clinical evidence remains inconclusive. First, studies differ in the timing of immunotherapy initiation, including induction, concurrent, and consolidation settings, as well as in ICI types, and consolidation duration, making direct comparisons difficult. Second, immune-related adverse events (irAEs), especially the overlapping risk of radiation pneumonitis and ICI-related pneumonitis, remain an important safety concern (17, 18). More importantly, not all patients benefit from consolidation immunotherapy, highlighting the need to identify patients who are most likely to benefit. PD-L1 is one of the most widely studied biomarkers for immunotherapy and has shown predictive value in advanced esophageal cancer. Some studies have suggested benefit regardless of PD-L1 status, while others have reported greater benefit in patients with high PD-L1 expression; however, these findings remain inconsistent and require validation (19–22).

Therefore, this study used real-world data from three centers between July 2019 and December 2024 to evaluate the efficacy and safety of PD-1 inhibitor consolidation therapy after dCRT in patients with locally advanced ESCC. This study also explored whether PD-L1 expression, assessed using the CPS, was associated with treatment benefit.

## 2 Materials and methods

### 2.1 Patients and study design

This multicenter retrospective cohort study included patients with locally advanced ESCC who were treated in the Department of Radiation Oncology at Zhenjiang First People’s Hospital, Danyang Hospital of Traditional Chinese Medicine, and Yangzhong People’s Hospital between July 2019 and December 2024. The follow-up cutoff and data cutoff date was December 2025. This study was approved by the Ethics Committees of all three participating institutions (Zhenjiang First People’s Hospital: SQK-20240111-W; Yangzhong People’s Hospital: L202620; Danyang Hospital of Traditional Chinese Medicine: 2026-DYZYY-005).

Patients were eligible for inclusion if they met the following criteria: (1) pathologically or cytologically confirmed ESCC; (2) ESCC clinically staged as T0-4, N0-3, M0-1 according to the American Joint Committee on Cancer (AJCC) 8th edition staging system for esophageal cancer, with M1 disease limited to supraclavicular lymph node metastasis; patients who were unsuitable for or refused surgical treatment; (3) received dCRT with a radiotherapy dose ≥50.4 Gy and completed 2 cycles of concurrent chemotherapy; (4) an Eastern Cooperative Oncology Group (ECOG) performance status (ECOG PS) of 0–1; (5) completion of dCRT without progression at the first post-treatment assessment; (6) sufficient fitness to be considered for consolidation immunotherapy; (7) complete clinical data and follow-up information available. (8) no prior or concurrent receipt of immunotherapy or other antineoplastic biologic agents.

Patients were excluded if they met any of the following criteria: (1) prior history of other malignant tumors; (2) prior or concurrent receipt of immunotherapy or other antineoplastic biologic agents; (3) presence of active autoimmune diseases or interstitial lung disease; (4) did not complete dCRT or did not complete 2 cycles of concurrent chemotherapy; (5) progression before the first post-treatment assessment; (6) underwent surgery after concurrent chemoradiotherapy; (7) incomplete clinical data; (8) expected survival <3 months; (9) incomplete follow-up records.

All patients were staged according to the AJCC 8th edition staging system. According to this system, supraclavicular lymph node metastasis is classified as M1 disease. In the present study, M1 disease was limited to supraclavicular lymph node metastasis, and no patient had other distant nodal or organ metastases. These patients were included in the present study based on the following considerations: (1) anatomically, the supraclavicular lymph nodes, particularly the ipsilateral nodes, are contiguous with the mediastinal nodal regions. In clinical practice, their involvement is generally regarded as an extension of locoregional disease rather than hematogenous dissemination; Supraclavicular lymph node involvement was defined as lymph nodes located above the clavicle, which can be covered by the extended curative-intent radiation field. (2) from a therapeutic perspective, these patients typically receive the same curative-intent dCRT as patients with locally advanced disease. In the present study, supraclavicular nodal involvement classified as M1 was present in 14 patients in the non-immunotherapy group and 11 patients in the immunotherapy group. The term “locally advanced ESCC” in this study is an operational cohort definition based on curative-intent treatment and radiation-field coverage; it does not represent a reclassification of the AJCC staging system.

Based on whether patients received consolidation immunotherapy within 4–8 weeks after completion of dCRT, they were divided into the consolidation immunotherapy group and the non-immunotherapy group. The index date was defined as the start of consolidation. OS and PFS were calculated from the index date. The treatment-assignment date was defined as the start of consolidation immunotherapy in the immunotherapy group and the start of consolidation chemotherapy in the non-immunotherapy group. The median interval from dCRT completion to consolidation initiation was 4.4 weeks (range, 3.7–6.0 weeks) in the consolidation immunotherapy group and 4.3 weeks (range, 3.9–7.0 weeks) in the non-immunotherapy group.

### 2.2 Treatment protocol

Concurrent Chemoradiotherapy: All patients received definitive intensity-modulated radiation therapy (IMRT) or volumetric modulated arc therapy (VMAT). The prescribed radiation dose ranged from 50.4 to 60 Gy in 25–30 fractions. Concurrent chemotherapy regimens mainly consisted of Taxane plus platinum (TP), or fluorouracil plus platinum (FP), administered every 3–4 weeks per cycle. Detailed doses, cycles, and completion rates are provided in **Supplementary Table 8**. Dose reductions or delays were made at the treating physician’s discretion.

All enrolled patients received dCRT and were scheduled to receive two cycles of chemotherapy during radiotherapy. At the first post-dCRT response assessment, performed 4 weeks after completion of radiotherapy, patients without disease progression received either consolidation chemotherapy alone (2 cycles) or consolidation chemotherapy combined with a PD-1 inhibitor. All patients in both groups received consolidation chemotherapy after dCRT. The choice of subsequent treatment was based on clinical assessment. The potential risks and benefits of each treatment option were fully discussed with the patients, and written informed consent was obtained. After consolidation chemotherapy, patients in the non-immunotherapy group underwent routine follow-up, whereas patients in the immunotherapy group continued PD-1 inhibitor maintenance therapy until disease progression, unacceptable toxicity, or a prespecified maximum duration of 2 years. Patients who received consolidation chemotherapy were included in the non-immunotherapy group. Those who received consolidation chemotherapy combined with a PD-1 inhibitor were included in the consolidation immunotherapy group.

Consolidation Immunotherapy: The PD-1 inhibitors used included camrelizumab (200 mg Q3W), tislelizumab (200 mg Q3W), sintilimab (200 mg Q3W), serplulimab (3 mg/kg Q2W), toripalimab (240 mg Q3W) or pembrolizumab (200 mg Q3W). Treatment was continued for up to 2 years or until disease progression, unacceptable toxicity, or treatment discontinuation. Due to the limited sample size of each drug subgroup (tislelizumab, *n* = 16; sintilimab, *n* = 11; camrelizumab, *n* = 9; serplulimab, *n* = 5; toripalimab, *n* = 3; pembrolizumab, *n* = 1; **Supplementary Table 8**), efficacy comparison or sensitivity analysis by agent type could not be performed.

### 2.3 PD-L1 assessment

Among the 96 enrolled patients, evaluable pretreatment tumor biopsy specimens were available in 38 patients, all from Zhenjiang First People’s Hospital. PD-L1 expression was assessed using immunohistochemistry (IHC). Immunohistochemical staining was performed using the E1L3N rabbit monoclonal antibody on the Leica Bond platform. Two experienced pathologists who were blinded to clinical outcomes independently evaluated the stained slides. Discordant cases were resolved by consensus, with a third senior pathologist consulted when necessary. The Combined Positive Score (CPS) was calculated as follows: (number of PD-L1-positive tumor cells, lymphocytes, and macrophages)/total number of viable tumor cells × 100. Based on previous studies and guideline recommendations, a CPS cutoff of 10 was used for stratification (23, 24). CPS ≥10 was defined as high PD-L1 expression, whereas CPS <10 was defined as low PD-L1 expression. PD-L1 testing was performed after patient enrollment was completed, using archived pretreatment tumor tissue, between January 2026 and February 2026.

### 2.4 Follow-up and assessment

Acute toxicities were assessed weekly during dCRT and consolidation therapy and monitored for 4 weeks after treatment completion. The initial post-dCRT response assessment was performed 4 weeks after completion of dCRT. Subsequent assessments were performed every 3 months during the first 2 years and every 6 months thereafter until 5 years. Imaging assessments included barium esophagography and contrast-enhanced CT of the chest and abdomen. When a complete response was suspected or the imaging findings were indeterminate, PET-CT or endoscopy was performed for confirmation.

Tumor response was evaluated according to the Response Evaluation Criteria in Solid Tumors (RECIST) version 1.1 and classified as complete response (CR), partial response (PR), stable disease (SD), or progressive disease (PD). ORR was defined as the proportion of patients who achieved CR or PR after consolidation allocation, and the disease control rate (DCR) was defined as the proportion of patients who achieved CR, PR, or SD after consolidation allocation. Response at the landmark was used as the baseline for post-consolidation response assessment.

To address immortal-time and guarantee-time biases, survival analyses used a landmark approach. The landmark was set at the start of consolidation, defined as the first day of consolidation chemotherapy in the non-immunotherapy group and the first day of concurrent consolidation chemotherapy plus PD-1 inhibitor in the immunotherapy group. PFS was defined as the time from the landmark to the first occurrence of PD or death from any cause. OS was defined as the time from the landmark to death from any cause. Patients who experienced progression or death before the landmark would have been excluded from the primary analysis. In our cohort, no patient experienced progression or death before the landmark; therefore, no patient was excluded. Treatment groups were defined according to receipt of immunotherapy at the landmark.

### 2.5 Safety analysis

Toxicity records were reviewed through December 2025. Adverse events were graded according to the National Cancer Institute Common Terminology Criteria for Adverse Events (CTCAE) version 5.0. For the safety comparison, TRAEs were analyzed over a prespecified common risk period of 12 months from the initiation of dCRT in both groups, ensuring equivalent observation periods. TRAEs were analyzed at the patient level; for each TRAE category, each patient was counted once, and only the highest grade was recorded. For example, if a patient experienced grade 1, grade 2, and grade 3 leukopenia during treatment, this patient would be counted only once in the “leukopenia” category, with the highest grade (grade 3) recorded. TRAEs were categorized as hematologic TRAEs, radiation pneumonitis, and immune-related TRAEs. Acute toxicity was defined as TRAEs occurring during dCRT and within 3 months after its completion; late radiation toxicity was defined as radiation pneumonitis occurring >3 months after dCRT. Immune-related TRAEs were defined as events occurring after PD-1 initiation, including those after treatment discontinuation. Because the observation periods were identical, duration-adjusted incidence rates were not required. The column totals represent the sum of patients across all TRAE categories, not the total number of patients. A single patient may contribute to more than one category; however, within each category, each patient is counted only once at the highest grade.

### 2.6 Differentiation between radiation pneumonitis and immune-related pneumonitis

For all patients presenting with new pulmonary imaging abnormalities, infection and tumor progression were first excluded through sputum culture, hematological workup, and clinical evaluation. Subsequently, a chest radiologist and an oncologist jointly reviewed the imaging findings and adjudicated the predominant etiology of pneumonitis based on the following four integrated dimensions:

1. Lesion distribution: lesions confined to the radiation field favored radiation pneumonitis (RP), whereas diffuse or symmetric distribution outside the radiation field favored checkpoint inhibitor pneumonitis (CIP) (25);
2. Timing of onset: with reference to the chronological relationship between radiotherapy completion and immunotherapy administration, RP typically occurred 1–6 months after radiotherapy, while CIP could arise at any time during the immunotherapy course (26);
3. Imaging characteristics: CIP commonly presented with organizing pneumonia-like or nonspecific interstitial pneumonia-like patterns with possible migratory distribution, whereas RP typically manifested as ground-glass opacities, consolidation, and traction bronchiectasis confined to the radiation field (27);
4. Response to corticosteroids and recurrence patterns: both conditions responded to corticosteroids, but CIP tended to have a higher recurrence tendency (26).

In cases with overlapping features or diagnostic uncertainty, final classification was reached by consensus through multidisciplinary team (MDT) discussion (27–29). In our study, this consensus discussion involved at least two radiation oncologists and two radiologists. A case-by-case summary of all pneumonitis events, including onset time after dCRT, onset time after PD-1 initiation, radiological distribution, grade, management, and outcome, is provided in **Supplementary Table 10**.

### 2.7 Statistical analysis

Statistical analyses were performed using SPSS 27.0 and R software 4.6.0. Continuous variables were presented as mean ± standard deviation or median (range) and compared using the *t*-test or Mann-Whitney U test. Categorical variables were expressed as frequencies with percentages (%) and compared using the chi-square (*χ*²) test or Fisher’s exact test when expected counts were < 5. To minimize confounding by baseline imbalances inherent to the retrospective design, we performed IPTW as a sensitivity analysis. Propensity scores were estimated using a multivariable logistic regression model that included age, sex, ECOG performance status, tumor differentiation, tumor location, and T/N/M stages, chemotherapy, radiotherapy dose, Charlson Comorbidity Index (CCI), post-dCRT response (CR/PR vs SD), and chemotherapy completion. The CCI was calculated based on the presence of comorbid conditions. In our cohort, the CCI ranged from 0 to 2; no patient had a CCI >2. Post-dCRT response was defined as the best response at the first post-dCRT assessment before consolidation initiation. Because CR is difficult to reliably assess after dCRT in esophageal cancer, patients with CR or PR were combined into a single category (CR/PR) for the propensity score model. BMI was not included because all values were within the normal range and well balanced (SMD = 0.08).

Stabilized IPTW weights were generated with the average treatment effect as the estimand. Covariate balance before and after weighting was assessed using standardized mean differences (SMDs), with an absolute SMD < 0.1 indicating adequate balance. SMD between 0.1 and 0.25 were considered acceptable in observational studies (30, 31). Detailed IPTW diagnostics, including propensity score distributions, weight distribution, maximum weights, effective sample size, and trimming/truncation analyses, are provided in **Supplementary Tables 1**-**5**; **Supplementary Figures 1**, **2**. The log-rank test was used for unweighted survival comparisons. Cox proportional hazards models were used to estimate hazard ratios (*HRs*) with 95% confidence interval (*CIs*) for both unweighted and IPTW-weighted analyses (32); weighted models incorporated IPTW as weights and robust standard errors. All tests were two-sided, and *P* < 0.05 was considered statistically significant.

## 3 Results

### 3.1 Patient selection

Between July 2019 and December 2024, a total of 495 patients with locally advanced ESCC received first-line dCRT at three center hospitals. Among them, 96 patients met the inclusion criteria and were included in the study (**Figure 1**). Of 495 screened patients, 399 were excluded; the detailed exclusion categories and counts are shown in **Figure 1**. The pre-consolidation interval was comparable between groups (4.4 vs. 4.3 weeks). OS and PFS were calculated from the start of consolidation.

**Figure 1:**
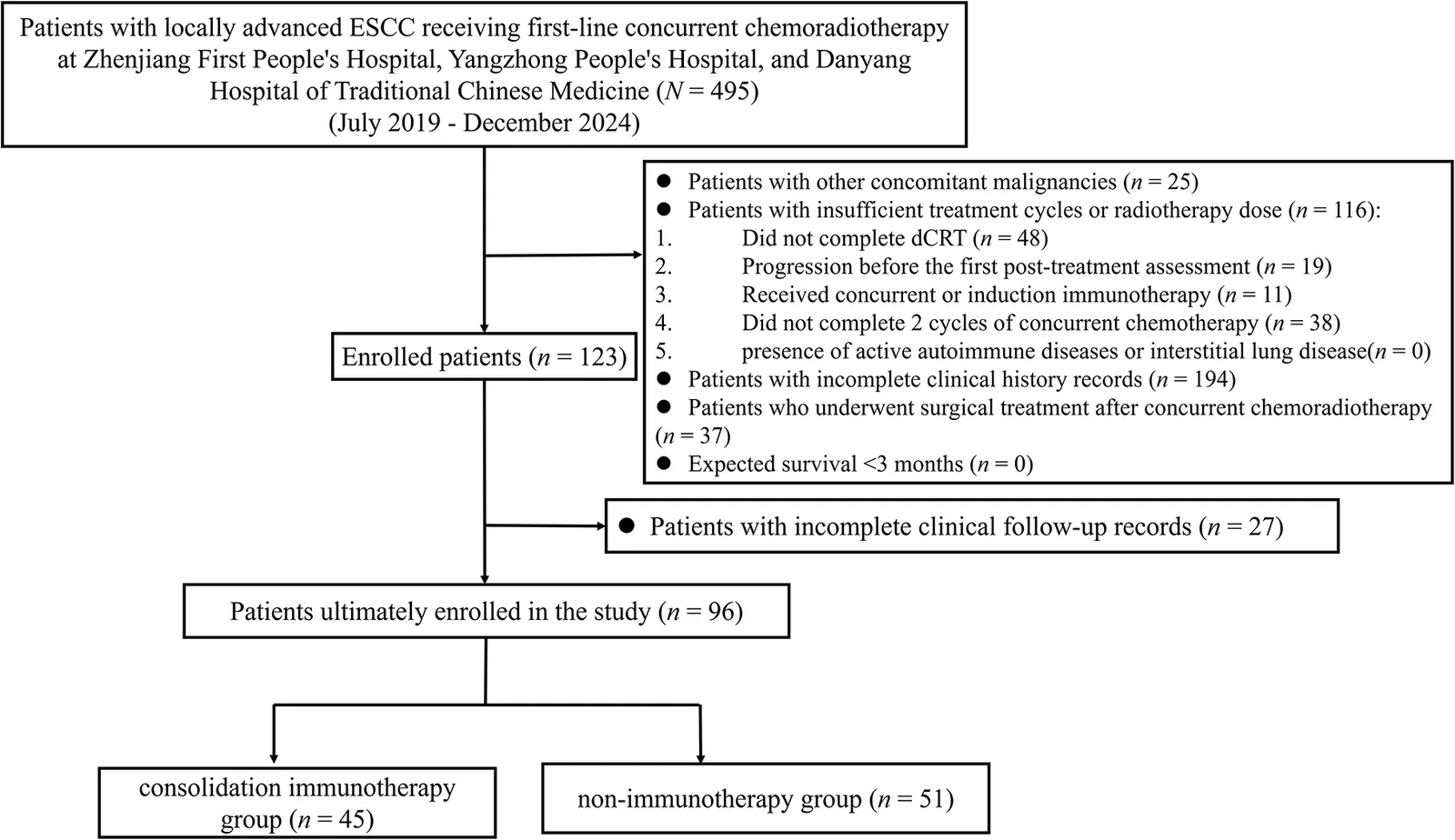
Patient screening flowchart.

### 3.2 Clinical and treatment characteristics

A total of 96 patients were diagnosed with locally advanced unresectable ESCC by gastroscopy and imaging examinations, including 51 patients in the non-immunotherapy group and 45 patients in the consolidation immunotherapy group.

The median time from dCRT to the first PD-1 dose was 31 days (IQR 29–33). The median number of PD-1 cycles was 8 (IQR 6–12), and the median treatment duration was 6.8 months (IQR 5.1–12.4).

In the non-immunotherapy group, the concurrent and consolidation chemotherapy regimens were TP in 38 patients (74.5%) and FP in 13 patients (25.5%); 46 patients (90.2%) completed 4 cycles of chemotherapy. In the consolidation immunotherapy group, the concurrent and consolidation chemotherapy regimens were TP in 30 patients (66.7%) and FP in 15 patients (33.3%); 43 patients (95.6%) completed 4 cycles of chemotherapy, and 42 patients (93.3%) completed at least 4 cycles of immunotherapy. The median number of concurrent and consolidation chemotherapy cycles was 2 (IQR 2–2) in both groups. Importantly, the two groups were comparable in chemotherapy regimens (TP/FP) and cycles; the only difference was the addition of consolidation PD-1 inhibitor in the immunotherapy group. Discontinuation of PD-1 inhibitor was due to disease progression in 16 patients (35.6%), patient preference in 19 patients (42.2%), completion of 2-year treatment in 3 patients (6.7%), and ongoing treatment at last follow-up in 7 patients (15.6%). Detailed treatment exposure data are provided in **Supplementary Table 8**.

Baseline clinical characteristics of the two groups are summarized in **Table 1**. The two groups were generally well balanced with respect to baseline clinical characteristics.

**Table 1:** Baseline characteristics of patients in the two groups.

Characteristic		Non-immunotherapy group (*n* = 51)	Consolidation immunotherapy group (*n* = 45)	*χ*² value	*P* value
Age	<65 years	21 (41.2)	22 (48.9)	0.575	0.44
	≥65 years	30 (58.8)	23 (51.1)		
Sex	Female	15 (29.4)	9 (20.0)	1.129	0.29
	Male	36 (70.6)	36 (80.0)		
ECOG	0	33 (64.7)	31 (68.9)	0.188	0.664
	≥1	18 (35.3)	14 (31.1)		
CCI	0	43 (84.3)	31 (68.9)	–	0.1741
	1	6 (11.8)	12 (26.7)		
	2	2 (3.9)	2 (4.4)		
Differentiation	High	13 (25.5)	8 (17.8)	0.975	0.614
	Top	22 (43.1)	23 (51.1)		
	Low	16 (31.4)	14 (31.1)		
Tumor location	Cervical	3 (5.9)	2 (4.4)	–	0.867
	Upper thoracic	7 (13.7)	4 (8.9)		
	Top thoracic	26 (51.0)	26 (57.8)		
	Lower thoracic	15 (29.4)	13 (28.9)		
T stage	T0	0 (0.0)	0 (0.0)	–	0.834
	T1	3 (5.9)	2 (4.4)		
	T2	10 (19.6)	6 (13.3)		
	T3	32 (62.7)	32 (71.2)		
	T4	6 (11.8)	5 (11.1)		
N stage	N0	9 (17.6)	9 (20.0)	–	0.964
	N1	24 (47.1)	19 (42.2)		
	N2	15 (29.4)	14 (31.1)		
	N3	3 (5.9)	3 (6.7)		
M stage	M0	37 (72.5)	34 (75.6)	0.112	0.738
	M1	14 (27.5)	11 (24.4)		
Chemotherapy	Taxane + platinum	38 (74.5)	30 (66.7)	0.712	0.399
	Fluorouracil + platinum	13 (25.5)	15 (33.3)		
Chemotherapy completion	4	46 (90.2)	43 (95.6)	–	0.442
	<4	5 (9.8)	2 (4.4)		
Radiotherapy dose	50.4 Gy	33 (64.7)	26 (57.8)	0.484	0.486
	>50.4 Gy	18 (35.3)	19 (42.2)		
Post-dCRT response	SD	18 (35.3)	19 (42.2)	0.236	0.627
	PR	33 (64.7)	26 (57.8)		
PD-L1 testing available	Yes	22 (43.1)	16 (35.6)	0.575	0.448
	No	29 (56.9)	29 (64.4)		
Immunotherapy				–	–
Sintilimab		–	11 (24.4)		
Camrelizumab		–	9 (20.0)		
Tislelizumab		–	16 (35.6)		
Serplulimab		–	5 (11.1)		
Toripalimab		–	3 (6.7)		
Pembrolizumab		–	1 (2.2)		

Data are presented as *n* (%). χ², chi-square test; “-” indicates that Fisher’s exact test was used instead of the *χ*² test.

### 3.3 Covariate balance assessment

Baseline characteristics of the unweighted cohort are presented in **Table 1**, and covariate balance before and after IPTW is illustrated in **Figure 2**. The balance of covariates was assessed using standardized mean differences (SMDs), with an SMD<0.1 considered indicative of acceptable balance. Prior to IPTW, substantial imbalances were observed for several covariates, particularly CCI (SMD = 0.385 for CCI_1 and −0.371 for CCI_0), sex (SMD = 0.220), and chemotherapy completion (SMD = −0.209). After applying IPTW weights, all covariates achieved SMDs below the prespecified threshold of 0.1 except for moderate differentiation (SMD = 0.132; **Supplementary Table 5**), which fell within the range of 0.1-0.25 that has been considered acceptable in observational studies. As a sensitivity analysis, we additionally adjusted for differentiation in the outcome model (IPWRA, doubly robust estimation), and the treatment effect remained consistent with the primary analysis (**Supplementary Table 15**, **Supplementary Figure 4**). The Love plot (**Figure 2**) visualizes the SMDs before and after weighting, demonstrating that IPTW substantially reduced baseline differences between the treatment groups.

**Figure 2:**
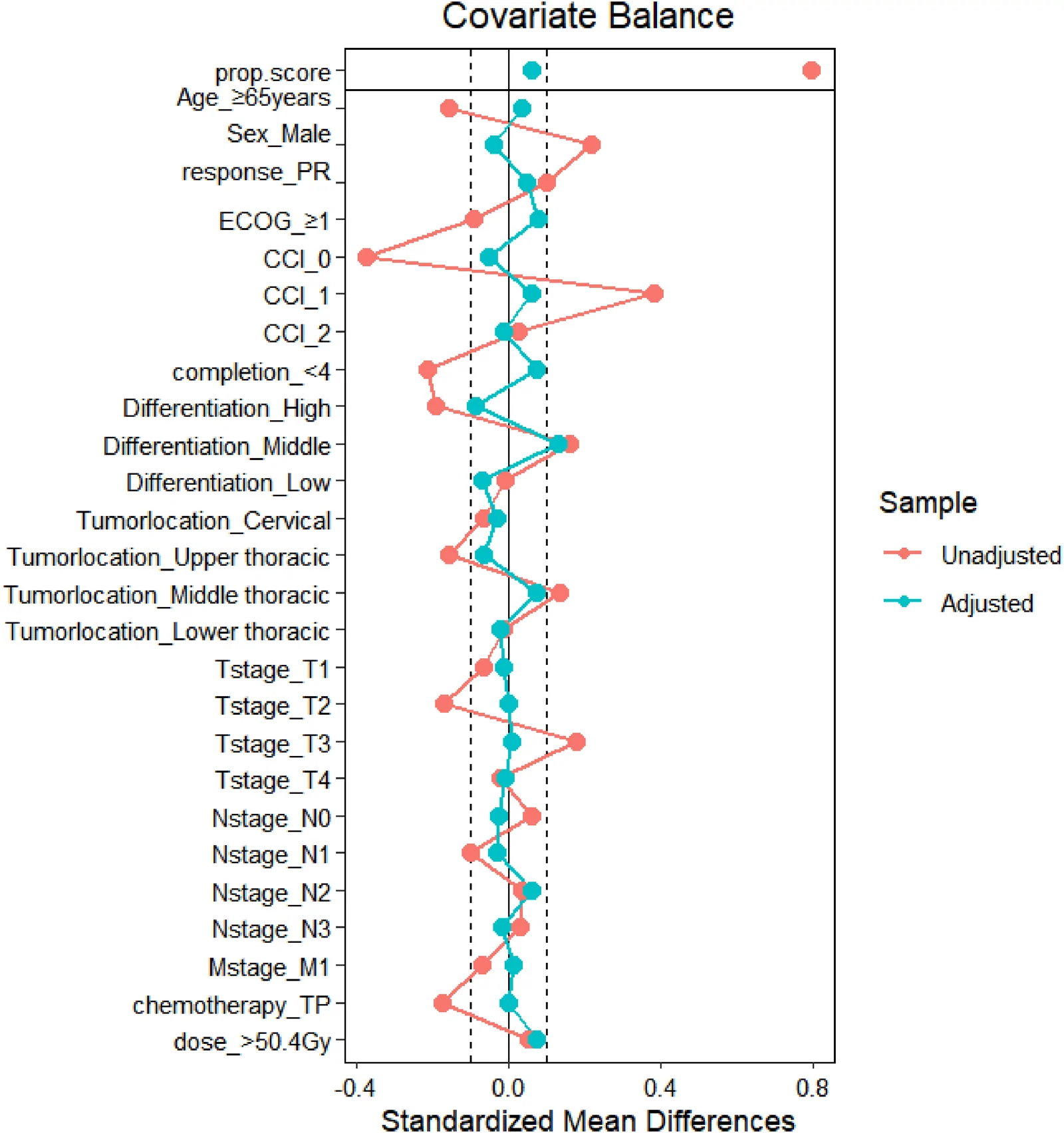
Love plot of standardized mean differences (SMDs) for all covariates before and after IPTW.

### 3.4 Short-term efficacy

#### 3.4.1 Tumor response outcomes

The median follow-up duration for the entire cohort was 29.4 months. Tumor response outcomes are summarized in **Table 2**. Among the 45 patients in the consolidation immunotherapy group, imaging evaluations revealed 3 cases of CR, 8 cases of SD, 24 cases of PR, and 10 cases of PD. The overall ORR was 60.0% (95% *CI* 44.3-74.3), and the overall DCR was 77.8% (95% *CI* 62.9-88.8).

**Table 2:** Comparison of short-term efficacy, ORR, and DCR between the two groups.

Variable	Consolidation immunotherapy group (*n* = 45)	Non-immunotherapy group (*n* = 51)	*χ*² value	*P* value
Overall ORR, % (95% *CI*)	60.0% (44.3-74.3) (27/45)	27.4% (15.9-41.7) (14/51)	10.351	0.001
Overall DCR, % (95% *CI*)	77.8% (62.9-88.8) (35/45)	52.9% (38.5-67.1) (27/51)	6.447	0.011

Among the 51 patients in the non-immunotherapy group, imaging evaluations revealed 1 case of CR, 13 cases of SD, 13 cases of PR, and 24 cases of PD. The overall ORR was 27.4% (95% *CI* 15.9–41.7), and the overall DCR was 52.9% (95% *CI* 38.5–67.1).

Post-consolidation response shift according to landmark disease status is summarized in **Supplementary Tables 6**, **7**; Improvement occurred in 15/45 (33.3%) vs. 9/51 (17.6%), and progression in 10/45 (22.2%) vs. 24/51 (47.1%) (*χ*² test, *P* = 0.048).

Among all 96 patients, the 1-year PFS and OS rates were 63.1% and 97.8% in the consolidation immunotherapy group, respectively, compared with 42.9% and 79.9% in the non-immunotherapy group.

#### 3.4.2 Survival outcomes-PFS and OS

Kaplan-Meier curves for PFS and OS before and after IPTW adjustment are shown in **Figure 3**. Median PFS and OS were calculated from the landmark (start of consolidation).

**Figure 3:**
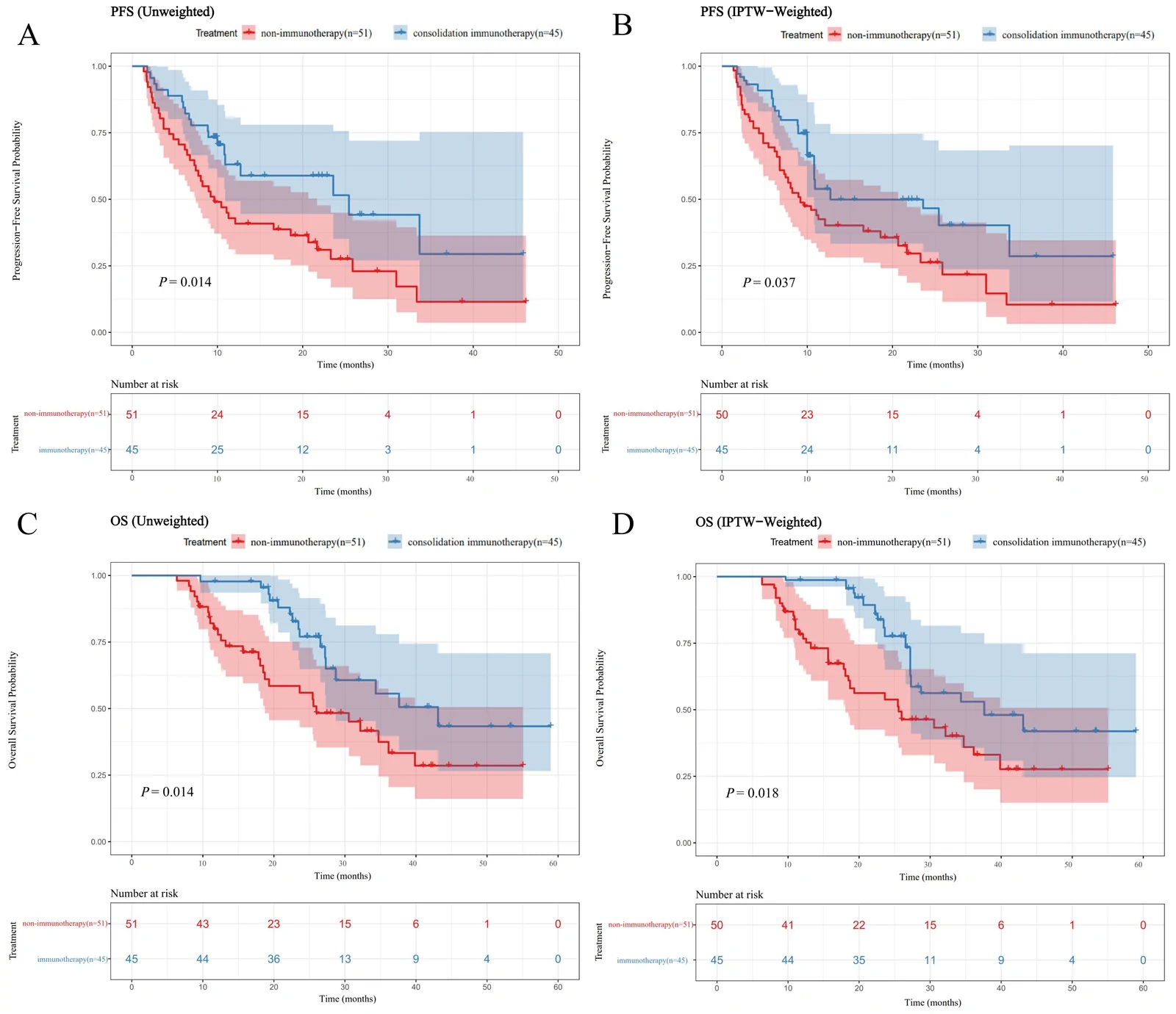
Kaplan-Meier curves for PFS and OS in the overall cohort, before and after IPTW adjustment. **(A)** PFS, unweighted; **(B)** PFS, IPTW-weighted; **(C)** OS, unweighted; **(D)** OS, IPTW-weighted. Red, non-immunotherapy group (*n* = 51); blue, consolidation immunotherapy group (*n* = 45). Shaded areas, 95% *CIs*. *P* values were calculated using the log-rank test for unweighted analyses and weighted Cox models for IPTW-adjusted analyses.

##### 3.4.2.1 Survival outcomes-PFS

Before IPTW adjustment, the median PFS was 25.4 months (95% *CI* 10.9–NR) in the consolidation immunotherapy group versus 9.6 months (95% *CI* 7.5–21.7) in the non-immunotherapy group. Kaplan-Meier analysis demonstrated a statistically significant difference in PFS between the two groups (*HR* = 0.507, 95% *CI* 0.292–0.880; log-rank *P* = 0.014; Cox *P* = 0.016) (**Figure 3A**).

After IPTW adjustment, the median PFS was 12.7 months (95% *CI* 10.8–NR) in the consolidation immunotherapy group versus 9.2 months (95% *CI* 6.8–20.7) in the non-immunotherapy group. Kaplan-Meier analysis demonstrated a statistically significant difference in PFS between the two groups (*HR* = 0.550, 95% *CI* 0.313–0.965; Cox *P* = 0.037) (**Figure 3B**).

##### 3.4.2.2 Survival outcomes-OS

Before IPTW adjustment, the median OS was 43.1 months (95% *CI* 28.7–NR) versus 26.0 months (95% *CI* 18.7–NR) in the non-immunotherapy group (*HR* = 0.469, 95% *CI* 0.253–0.870; log-rank *P* = 0.014; Cox *P* = 0.016) (**Figure 3C**).

After IPTW adjustment, the median OS was 37.6 months (95% *CI* 27.2–NR) versus 25.6 months (95% *CI* 18.1–NR) (*HR* = 0.465, 95% *CI* 0.247–0.877; Cox *P* = 0.018) (**Figure 3D**). Detailed event numbers, median follow-up, proportional hazards assumption testing, time-varying coefficient Cox model, and RMST analyses are provided in **Supplementary Tables 12**, **13**.

In a sensitivity analysis excluding patients with M1 disease (M0 cohort: non-immunotherapy group, *n* = 37; consolidation immunotherapy group, *n* = 34), the results were consistent with the primary analysis (median PFS: 11.1 vs. 25.4 months, *HR* = 0.52, 95% *CI* 0.28–0.98, log-rank *P* = 0.038; median OS: 26.0 months vs. not reached, *HR* = 0.37, 95% *CI* 0.17–0.81, log-rank *P* = 0.010; **Supplementary Figure 3**).

#### 3.4.3 PD-L1 stratified survival analysis

##### 3.4.3.1 Overall characteristics of the PD-L1 evaluable population

Among the 96 enrolled patients, 38 had evaluable pretreatment tumor tissue and underwent PD-L1 testing (non-immunotherapy group, *n* = 22; consolidation immunotherapy group, *n* = 16). High PD-L1 expression (CPS ≥10) was observed in 13 patients (34.2%), and low PD-L1 expression (CPS <10) in 25 patients (65.8%). The two groups were comparable in baseline characteristics (**Supplementary Table 11**).

Kaplan-Meier curves for PFS and OS in the PD-L1 evaluable cohort are shown in **Figures 4A, B**. The median PFS was not reached (95% *CI* 6.9–NR) in the consolidation immunotherapy group versus 7.9 months (95% *CI* 6.2–12.1) in the non-immunotherapy group (*HR* = 0.423, 95% *CI* 0.178–1.003; log-rank *P* = 0.044; Cox *P* = 0.051). The median OS was 43.1 months (95% *CI* 28.7–NR) versus 19.3 months (95% *CI* 18.1–NR) (*HR* = 0.400, 95% *CI* 0.151–1.055; log-rank *P* = 0.056; Cox *P* = 0.064);. The 95% confidence intervals crossed 1, and the Cox *P* values were borderline (PFS: 0.0509; OS: 0.0641). The 95% confidence intervals for the *HRs* crossed 1 in both PFS and OS, indicating that the treatment associations did not reach statistical significance. These results should be interpreted as exploratory only.

**Figure 4:**
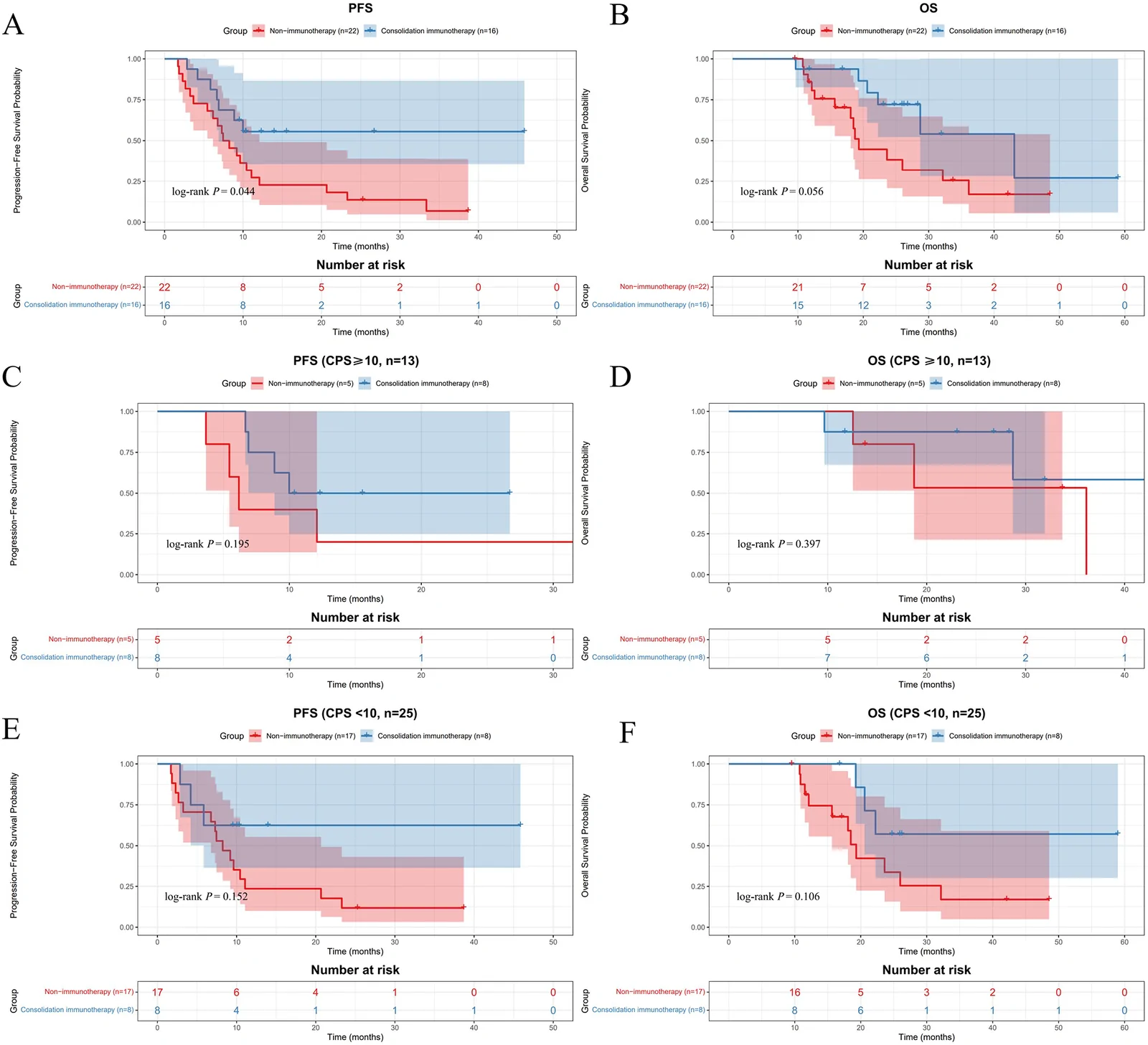
Kaplan-Meier curves for PFS and OS in the PD-L1 evaluable population and CPS subgroups. **(A)** PFS and **(B)** OS in the PD-L1 evaluable cohort (*n* = 38); **(C)** PFS and **(D)** OS in the CPS ≥10 subgroup (*n* = 13); **(E)** PFS and **(F)** OS in the CPS <10 subgroup (*n* = 25). Red, non-immunotherapy group; blue, consolidation immunotherapy group. Shaded areas indicate 95% confidence intervals.

##### 3.4.3.2 Survival analysis stratified by PD-L1 expression

Patients were stratified into two subgroups according to CPS: CPS ≥10 (high expression, *n* = 13) and CPS <10 (low expression, *n* = 25). Kaplan-Meier curves for PFS and OS in the two subgroups are shown in **Figures 4C–F**.

###### 3.4.3.2.1 PD-L1 high expression subgroup (CPS ≥10, n = 13)

In the CPS ≥10 subgroup (non-immunotherapy group, *n* = 5; consolidation immunotherapy group, n=8), the median PFS was 10.0 months (95% *CI* 8.9–NR) in the consolidation immunotherapy group versus 6.2 months (95% *CI* 5.5–NR) in the non-immunotherapy group (*HR* = 0.406, 95% *CI* 0.100–1.655; log-rank *P* = 0.195; Cox *P* = 0.209) (**Figure 4C**). The median OS was 43.1 months (95% *CI* 28.7–NR) versus 36.1 months (95% *CI* 18.7–NR) (*HR* = 0.467, 95% *CI* 0.077–2.829; log-rank *P* = 0.397; Cox *P* = 0.407) (**Figure 4D**). In the CPS ≥10 subgroup, the *HRs* for both PFS and OS were < 1; however, the 95% *CIs* crossed 1 and the *P* values were >0.05, indicating no statistically significant difference. Given the very small sample size (*n* = 13) and severely limited statistical power, these findings should be interpreted as exploratory and hypothesis-generating only.

###### 3.4.3.2.2 PD-L1 low expression subgroup (CPS <10, n = 25)

In the CPS <10 subgroup (non-immunotherapy group, *n* = 17; consolidation immunotherapy group, *n* = 8), the median PFS was not reached (95% *CI* 5.8–NR) in the consolidation immunotherapy group versus 8.2 months (95% *CI* 6.8–20.7) in the non-immunotherapy group (*HR* = 0.414, 95% *CI* 0.119–1.437; log-rank *P* = 0.152; Cox *P* = 0.165) (**Figure 4E**). The median OS was not reached (95% *CI* 20.6–NR) versus 19.3 months (95% *CI* 15.6–NR) (*HR* = 0.360, 95% *CI* 0.099–1.307; log-rank *P* = 0.106; Cox *P* = 0.121) (**Figure 4F**). In the CPS <10 subgroup, the *HRs* for both PFS and OS were < 1; however, the 95% *CIs* crossed 1 and the *P* values were > 0.05, indicating no statistically significant difference. Given the small sample size (*n* = 25) and limited statistical power, these results should be interpreted as exploratory and hypothesis-generating only.

Detailed event numbers, median follow-up, and results of the proportional hazards assumption testing for the PD-L1 evaluable cohort and CPS subgroups are provided in **Supplementary Table 14**.

#### 3.4.4 Safety analysis

TRAEs are summarized in **Table 3**. All 96 patients were included in the safety analysis. A total of 262 TRAEs were observed.

**Table 3:** Comparison of treatment-related adverse events between the two groups.

Event	Non-immunotherapy group (*n* = 51)		Consolidation immunotherapy group (*n* = 45)	
	Grade 1–2	Grade 3–4	Grade 1–2	Grade 3–4
	Patients, *n*	Patients, *n*	Patients, *n*	Patients, *n*
Hematologic TRAEs				
AST elevation	10	2	8	1
ALT elevation	14	1	12	0
Total bilirubin elevation	8	1	7	0
Platelet count decreased	21	4	15	3
White blood cell count decreased	35	5	25	3
Neutrophil count decreased	34	4	23	2
Radiation pneumonitis	6	1	4	2
Acute (≤3 months)	1	0	1	0
Late (>3 months)	5	1	3	2
Immune-related adverse events (irAEs)				
RCCEP	–	–	3	–
Rash/Pruritus	–	–	1	–
Immune myocarditis	–	–	0	–
Immune pneumonitis	–	–	2	–
Immune hypothyroidism	–	–	4	–
Immune hepatitis	–	–	0	–
Immune nephritis	–	–	0	–
Diarrhea/Colitis	–	–	1	–
Grade ≥3 TRAEs (patient level), % (95% *CI*) (*P* = 0.660)	23.5 (12.8–37.5)		17.8 (8.0–32.1)	

Data are presented as the number of patients (*n*) with the highest recorded grade for each TRAE category. For each category, each patient was counted once at the highest grade. Because a single patient may contribute to more than one category, the sum of patients across all categories exceeds the total number of patients. In the non-immunotherapy group, the column totals for grade 1–2 and grade 3–4 TRAEs were 128 and 18 (total 146); in the immunotherapy group, the corresponding totals were 94 and 11 (total 105). An additional 11 immune-related adverse events (irAEs) occurred in the immunotherapy group and are listed separately; the total number of TRAEs was therefore 262 (146 + 116). RCCEP, reactive cutaneous capillary endothelial proliferation. Grade ≥3 TRAEs at the patient level were observed in 23.5% (12/51) of the non-immunotherapy group and 17.8% (8/45) of the immunotherapy group (*P* = 0.660).

In the consolidation immunotherapy group (*n* = 45), 116 TRAEs occurred; the incidence of grade ≥3 TRAEs at the patient level was 17.8% (8/45). In the non-immunotherapy group (*n* = 51), 146 TRAEs occurred; the incidence of grade ≥3 TRAEs at the patient level was 23.5% (12/51) (*P* = 0.660).

Immune-related adverse events (irAEs) occurred only in the consolidation immunotherapy group. The most common irAEs were grade 1–2 thyroid dysfunction (4/11, 36.4%), reactive cutaneous capillary endothelial proliferation (3/11, 27.3%), and immune-related pneumonitis (2/11, 18.2%). No treatment-related deaths occurred in either group. TRAE-related clinical outcomes, including treatment discontinuation, steroid use, hospitalization, and pneumonitis resolution, are summarized in **Supplementary Table 9**.

In the non-immunotherapy group, 7 patients developed radiation pneumonitis (grade 1–2: 6; grade 3–4: 1); 6 cases occurred within 6 months of dCRT, and 1 case occurred at 6.5 months. In the immunotherapy group, 6 patients developed radiation pneumonitis (grade 1–2: 4; grade 3–4: 2), all occurring within 6 months of dCRT, and 2 patients developed immune pneumonitis (both grade 1–2), both occurring after PD-1 initiation and extending beyond the radiation field. A case-by-case summary of all pneumonitis events with adjudication is provided in **Supplementary Table 10**.

## 4 Discussion

This multicenter retrospective study evaluated the efficacy and safety of consolidation immunotherapy after dCRT in patients with locally advanced ESCC, using real-world data from three centers. IPTW was applied to balance measured baseline covariates and reduce confounding between the groups. Consolidation immunotherapy after dCRT was associated with longer PFS and OS and higher ORR and DCR. The incidence of grade ≥3 TRAEs did not differ significantly between the groups, and no apparent increase in severe late radiation-related toxicities was observed. Together, these findings provide preliminary real-world evidence on the feasibility of consolidation immunotherapy after dCRT in locally advanced ESCC. However, given the retrospective design, small sample size, and the negative primary endpoint of the phase 3 SKYSCRAPER-07 trial, these results should not be interpreted as supporting routine implementation of a “PACIFIC-like” strategy. Validation in larger, prospectively designed randomized trials is warranted.

From a mechanistic perspective, radiotherapy not only exerts direct cytotoxic effects on tumor cells but also induces immunogenic cell death. This process releases tumor antigens and danger signals including HMGB1 and ATP, thereby promoting dendritic cell (DC) maturation and effector T-cell priming (10). However, radiotherapy may also induce PD-L1 expression and other immunosuppressive changes in the tumor microenvironment. These changes may limit radiotherapy-induced antitumor immune responses (33, 34). Persistent antigen exposure and the post-radiotherapy tumor microenvironment may also promote progressive CD8+ T-cell dysfunction and exhaustion. Exhausted CD8+ T cells have distinct functional states. Progenitor exhausted subsets retain proliferative potential and may respond to PD-1 blockade. In contrast, terminally exhausted cells have limited capacity for functional recovery (35). PD-1 inhibitors may relieve inhibitory signaling in responsive exhausted T-cell subsets. This may sustain or partially restore antitumor activity after dCRT. Therefore, consolidation immunotherapy after dCRT may extend the immunomodulatory effects of radiotherapy and enhance local and systemic disease control (36, 37).

Several phase 3 trials are investigating immunotherapy combined with definitive CRT in locally advanced esophageal cancer. The KEYNOTE-975 trial is evaluating pembrolizumab plus dCRT versus placebo plus dCRT, with event-free survival and OS as the primary endpoints (38). The RATIONALE-311 trial was designed to evaluate tislelizumab plus concurrent CRT versus placebo plus concurrent CRT in patients with inoperable localized ESCC, with PFS as the primary endpoint. Full efficacy results have not yet been published (39). Both trials are evaluating whether the concurrent addition of immunotherapy to CRT improves survival outcomes. Evidence from unresectable stage III NSCLC suggests that the timing of immunotherapy in relation to CRT may be clinically relevant. The PACIFIC trial demonstrated durable survival benefits with consolidation durvalumab administered after concurrent CRT (40). In contrast, the PACIFIC-2 trial evaluated durvalumab initiated concurrently with CRT and continued as consolidation therapy but did not significantly improve PFS or OS compared with placebo (41). Against this background, our study focused on consolidation immunotherapy administered after completion of dCRT in patients with locally advanced ESCC.

Direct evidence for consolidation immunotherapy after dCRT in ESCC is emerging. At the 2025 ASCO Annual Meeting, Wang et al. reported the results of a single-arm phase 2 study of consolidation camrelizumab after dCRT. Median PFS and OS were not reached. The 1- and 2-year PFS rates were 81.3% and 60.6%, respectively, and the corresponding OS rates were 96.9% and 81.0% (42). Most adverse events were grade 1–2.

Further evidence was provided by the phase 3 SKYSCRAPER-07 trial, presented at the 2025 ESMO Congress (Abstract 2094O). The trial evaluated sequential consolidation immunotherapy in patients with unresectable ESCC without disease progression after dCRT (43). The combination of tiragolumab plus atezolizumab did not meet its primary endpoint of improved PFS versus placebo, and development of the tiragolumab combination was subsequently discontinued. Because the hierarchical testing requirements were not met, the atezolizumab monotherapy results should be interpreted as descriptive rather than confirmatory. However, results indicated a clinically meaningful improvement in PFS and OS with atezolizumab monotherapy versus placebo. Median OS was not reached with atezolizumab monotherapy, compared with 36.4 months in the placebo group (*HR*, 0.69, nominal *P* = 0.009). Median PFS was 29.1 with atezolizumab and 16.6 months with placebo (*HR*, 0.74, nominal *P* = 0.011). Based on these results, a regulatory application for atezolizumab as maintenance therapy following dCRT has been filed. Because the hierarchical testing requirements were not met, these findings should be interpreted as descriptive rather than confirmatory.

Several recent retrospective studies have also investigated consolidation immunotherapy after dCRT. Yu et al. conducted a real-world multicenter study and reported significantly longer OS in the dCRT + cICI group than in the dCRT group before sIPTW adjustment (median, 46.4 vs. 20.9 months; *P* = 0.02). PFS was also significantly longer in the dCRT + cICIs group (median, 27.4 vs. 12.9 months; *P* = 0.02). After sIPTW adjustment, the differences in OS and PFS were no longer statistically significant, although both outcomes remained numerically longer in the dCRT + cICIs group (44). These studies, however, differ in how they define the study population, particularly regarding the inclusion of patients with supraclavicular lymph node metastasis classified as M1 under the AJCC 8th edition. This issue is directly relevant to the interpretation of our cohort.

In this study, patients with supraclavicular lymph node metastasis classified as M1 under the AJCC 8th edition were included because all lesions could be covered by the curative-intent radiation field and because these patients received curative-intent dCRT. This approach is supported by the Japanese Esophageal Society (JES) classification, which differs from the AJCC system in the categorization of supraclavicular lymph node metastasis. The JES 11th edition included the supraclavicular lymph nodes (station 104) within the regional lymph nodes for thoracic esophageal cancer (45). The JES 12th edition further aligned with the UICC/AJCC system and reclassified supraclavicular lymph node metastasis as M1a, distinguishing it from other distant metastases (M1b) (46, 47). This classification reflects the clinical distinction between supraclavicular lymph node metastasis and widespread distant metastasis, and indicates that some of these patients may still be candidates for curative-intent treatment. Importantly, the present study used the AJCC 8th edition for staging and did not reclassify supraclavicular lymph node metastasis as regional disease; the JES classification is cited only to explain the background of curative-intent treatment in East Asian clinical practice.

Takahashi et al. described supraclavicular lymph node metastasis in ESCC as “potentially curable distant metastases” (48). However, that study was based mainly on neoadjuvant chemotherapy and surgical treatment and cannot directly demonstrate the efficacy of dCRT in these patients, nor does it change their M1 classification under the AJCC 8th edition. We therefore cite this study only as clinical background and do not draw further inferences from it. In addition, the multicenter real-world study by Yu et al. described above used a population definition similar to ours, including patients with locally advanced ESCC who received dCRT and limiting M1 disease to supraclavicular lymph node metastasis (44). This study provides a relatively direct real-world reference for curative-intent dCRT and subsequent consolidation immunotherapy in this population, but it is cited as a reference for similar study design and does not replace the AJCC staging criteria. A sensitivity analysis excluding patients with M1 disease showed that the direction of the treatment effect was consistent with the primary analysis (**Supplementary Figure 3**), supporting the robustness of our findings. With this population definition in mind, we next examined the treatment outcomes in our cohort.

In the present study, median PFS was 25.4 months (95% *CI* 10.9–NR) in the consolidation immunotherapy group (*n* = 45) versus 9.6 months (95% *CI* 7.5–21.7) in the non-immunotherapy group (*n* = 51) (*HR* = 0.507, 95% *CI* 0.292–0.880; log-rank *P* = 0.014; Cox *P* = 0.016). Median OS was 43.1 months (95% *CI* 28.7–NR) versus 26.0 months (95% *CI* 18.7–NR) (*HR* = 0.469, 95% *CI* 0.253–0.870; log-rank *P* = 0.014; Cox *P* = 0.016). After IPTW, median PFS remained longer in the consolidation immunotherapy group than in the non-immunotherapy group (12.7 vs. 9.2 months; *HR* = 0.550, 95% *CI* 0.313–0.965; Cox *P* = 0.037), as did median OS (37.6 vs. 25.6 months; *HR* = 0.465, 95% *CI* 0.247–0.877; Cox *P* = 0.018). Our results suggested an association between the addition of consolidation immunotherapy after dCRT and prolonged progression-free survival, higher ORR, and DCR. However, given the retrospective nature of this study and the potential for selection bias, these findings should be interpreted as exploratory, and no causal inference can be drawn. These survival outcomes are broadly consistent with those reported in previous studies, and provide further real-world evidence for this treatment strategy. However, residual confounding and treatment-selection bias cannot be excluded, and the magnitude of the observed associations may therefore have been overestimated.

In the setting of consolidation immunotherapy after dCRT for locally advanced ESCC, the role of PD-L1 remains uncertain. Radiotherapy can upregulate PD-L1 expression on tumor cells and immune cells within the tumor microenvironment (49). Consequently, pretreatment PD-L1 expression may not fully reflect PD-L1 status after dCRT. In our study, the *HRs* were < 1 in both the CPS ≥10 and CPS <10 subgroups, but the 95% *CIs* crossed 1 and the differences were not statistically significant. However, the subgroup analyses did not reach statistical significance for either PFS or OS in the CPS ≥10 subgroup (PFS: Cox *P* = 0.209; OS: Cox *P* = 0.407) or the CPS < 10 subgroup (PFS: Cox *P* = 0.165; OS: Cox *P* = 0.121). Only 38 patients had evaluable tumor tissue, including 16 in the consolidation immunotherapy group and 22 in the non-immunotherapy group. All specimens were obtained from a single center. Several baseline differences were observed between patients with and without evaluable tissue. Selection bias related to tissue availability therefore cannot be excluded. Numerical differences between the CPS subgroups do not establish predictive value. A predictive biomarker should identify a difference in treatment effect according to biomarker status, which requires a formal treatment-by-biomarker interaction test (50). Such an analysis could not be performed reliably in our study because of the small numbers of patients and events. The comparison between CPS ≥10 and CPS <10 was therefore considered an exploratory analysis of a possible prognostic association, and the treatment estimates within each CPS subgroup were descriptive. These analyses should therefore be considered hypothesis-generating and do not establish PD-L1 as a predictive biomarker for consolidation immunotherapy. The findings should not be used to guide treatment selection.

Liquid biopsy approaches such as exosomal PD-L1 analysis have been associated with response to anti-PD-1 therapy in melanoma and warrant further evaluation in ESCC (51). Composite models may provide additional information by integrating serial tissue and blood biomarkers with clinical and imaging features. However, these candidate biomarkers require stepwise validation in independent and prospective ESCC cohorts before clinical use (52). Importantly, the E1L3N antibody used in this study is not one of the principal companion-diagnostic assays for CPS-based treatment decisions. However, in ESCC, E1L3N has shown moderate to good concordance with the 22C3 companion diagnostic assay (Kappa 0.712–0.764 for CPS ≥10; overall percent agreement 0.853) (53, 54). In addition, 22C3, 28-8, and SP263 have demonstrated high concordance with each other in ESCC (Kappa 0.66–0.80 at CPS ≥10) (55). These findings suggest that E1L3N may be a reasonable alternative for exploratory analyses. Nevertheless, assay validation and concordance testing were not performed in our own cohort, and the PD-L1 results should be interpreted with caution. Due to the limited sample size (*n* = 38), a formal treatment-by-PD-L1 interaction test was not adequately powered and was not performed.

Regarding safety, the incidence of grade ≥3 TRAEs in the consolidation immunotherapy group did not differ significantly from that in the non-immunotherapy group (17.8% vs. 23.5%; *P* = 0.660), and most irAEs were grade 1–2. Pneumonitis is of particular concern when immunotherapy is administered after thoracic chemoradiotherapy (14). Radiation pneumonitis occurred in 6/45 (13.3%) and 7/51 (13.7%) patients, and immune pneumonitis occurred in 2/45 (4.4%) and 0/51 (0%) patients, respectively. Grade ≥3 pneumonitis occurred in 2/45 (4.4%) and 1/51 (2.0%) patients. This finding may partly reflect the use of IMRT or VMAT and the sequential rather than concurrent administration of immunotherapy. However, this explanation remains speculative because of the retrospective study design. Nevertheless, careful monitoring of respiratory symptoms and imaging findings remains essential in clinical practice.

This study has several limitations. First, its retrospective and nonrandomized design introduces the possibility of treatment-selection bias. Although IPTW was used to balance measured baseline covariates, residual confounding from unmeasured factors cannot be excluded. These include socioeconomic status, reimbursement considerations, treatment center, calendar year, tumor length, and physician preference. Second, the analysis was restricted to patients who completed dCRT and had no disease progression at the post-dCRT assessment. Accordingly, the findings apply primarily to patients eligible for consolidation therapy and may not be generalizable to all patients initiating dCRT. In addition, the inclusion of patients with supraclavicular lymph node metastasis classified as M1 under the AJCC 8th edition may affect generalizability and introduce selection bias, because AJCC and JES classify these patients differently and our cohort definition was operational rather than a reclassification of the AJCC system. Third, multiple PD-1 inhibitors were used. Except for tislelizumab, the number of patients receiving each agent was too small to permit meaningful agent-specific or sensitivity analyses. The findings should be interpreted as representing a heterogeneous PD-1 inhibitor strategy rather than evidence for any particular agent. Agent-specific analyses could not be performed due to the limited sample size of each drug subgroup. Differences in treatment duration and chemotherapy completion may also have affected the outcomes. Treatment exposure was heterogeneous, with a median of 8 PD-1 cycles (IQR 6–12) and a median treatment duration of 6.8 months (IQR 5.1–12.4). Fourth, the PD-L1 subgroup analysis was based on a small sample (*n* = 38) with few events, precluding multivariable adjustment. PD-L1 testing was performed at only one center, and archival tissue was not systematically preserved at other sites. Residual selection bias cannot be fully excluded. These findings are exploratory and require validation in larger cohorts. Fifth, the retrospective distinction between radiation pneumonitis and immune pneumonitis remains challenging. Although we applied a prespecified adjudication framework based on onset time and radiological distribution, with consensus discussion for overlapping cases, misclassification cannot be fully excluded. Finally, the overall sample size was limited, particularly for subgroup analyses. Therefore, our findings require validation in larger prospective randomized controlled trials.

Immunotherapy is being actively investigated for locally advanced ESCC (56). Beyond single-agent consolidation immunotherapy, strategies under investigation include concurrent immunotherapy with CRT and dual immune checkpoint blockade after dCRT (56, 57). Further studies are needed to optimize treatment sequence, radiotherapy dose and fractionation, and biomarkers for patient selection, and to identify patient subgroups that may derive greater benefit from concurrent or consolidation immunotherapy.

## 5 Conclusion

In this retrospective study, consolidation immunotherapy with PD-1 inhibitors after dCRT was associated with longer survival outcomes in patients with locally advanced ESCC. Grade ≥3 TRAEs were comparable between the two groups. Exploratory analyses of PD-L1 expression were inconclusive because of the small number of patients with evaluable tumor tissue. These findings require validation in prospective randomized trials before this strategy can be incorporated into routine clinical practice.

## Data Availability

The raw data supporting the conclusions of this article will be made available by the authors, without undue reservation.

## References

[1] Sung H Filho AM Laversanne M Ferlay J Siegel RL Soerjomataram I . Global cancer statistics 2024: GLOBOCAN estimates of incidence and mortality worldwide for 34 cancers in 186 countries. CA Cancer J Clin. (2026) 76:e70090. doi: 10.3322/caac.70090 42417444 PMC13343830

[2] Yang H Wang F Hallemeier CL Lerut T Fu J . Oesophageal cancer. Lancet. (2024) 404:1991–2005. doi: 10.1016/S0140-6736(24)02226-8 39550174

[3] Li Z Li J Yu C Hao J Sun J Chen Y . The global, regional, and national burden of early, middle, and late-onset esophageal cancer and trends from 1990 to 2021: a systematic analysis of the Global Burden of Disease Study 2021. J Thorac Dis. (2025) 17:8477–96. doi: 10.21037/jtd-2025-782 41229786 PMC12603433

[4] Ajani JA D’Amico TA Bentrem DJ Cooke D Corvera C Das P . Esophageal and esophagogastric junction cancers, version 2.2023, NCCN clinical practice guidelines in oncology. J Natl Compr Canc Netw. (2023) 21:393–422. doi: 10.6004/jnccn.2023.0019 37015332

[5] Cooper JS Guo MD Herskovic A Macdonald JS Martenson JA Jr Al-Sarraf M . Chemoradiotherapy of locally advanced esophageal cancer: long-term follow-up of a prospective randomized trial (RTOG 85-01). Radiation Therapy Oncology Group. JAMA. (1999) 281:1623–7. doi: 10.1001/jama.281.17.1623 10235156

[6] Shapiro J van Lanschot JJB Hulshof MCCM van Hagen P van Berge Henegouwen MI Wijnhoven BPL . Neoadjuvant chemoradiotherapy plus surgery versus surgery alone for oesophageal or junctional cancer (CROSS): long-term results of a randomised controlled trial. Lancet Oncol. (2015) 16:1090–8. doi: 10.1016/S1470-2045(15)00040-6 26254683

[7] Kato K Doki Y Chau I Xu J Wyrwicz L Motoyama S . Nivolumab plus chemotherapy or ipilimumab versus chemotherapy in patients with advanced esophageal squamous cell carcinoma (CheckMate 648): 29-month follow-up from a randomized, open-label, phase III trial. Cancer Med. (2024) 13:e7235. doi: 10.1002/cam4.7235 38716626 PMC11077338

[8] Xu J Kato K Hubner R Park SR Kojima T Ishihara R . First-line tislelizumab plus chemotherapy for esophageal squamous cell carcinoma with programmed death-ligand 1 expression ≥ 1%: A retrospective analysis of RATIONALE-306. Adv Ther. (2025) 42:2269–84. doi: 10.1007/s12325-025-03115-9 40075024 PMC12006250

[9] Luo H Lu J Bai Y Mao T Wang J Fan Q . Effect of camrelizumab vs placebo added to chemotherapy on survival and progression-free survival in patients with advanced or metastatic esophageal squamous cell carcinoma: the ESCORT-1st randomized clinical trial. JAMA. (2021) 326:916–25. doi: 10.1001/jama.2021.12836 34519801 PMC8441593

[10] Golden EB Frances D Pellicciotta I Demaria S Helen Barcellos-Hoff M Formenti SC . Radiation fosters dose-dependent and chemotherapy-induced immunogenic cell death. Oncoimmunology. (2014) 3:e28518. doi: 10.4161/onci.28518 25071979 PMC4106151

[11] Sun JM Shen L Shah MA Enzinger P Adenis A Doi T . Pembrolizumab plus chemotherapy versus chemotherapyalone for first-line treatment of advanced oesophageal cancer (KEYNOTE-590): a randomised, placebo-controlled, phase 3 study. Lancet. (2021) 398:759–71. doi: 10.1016/S0140-6736(21)01234-4 34454674

[12] Dagar G Gupta A Shankar A Chauhan R Macha MA Bhat AA . The future of cancer treatment: combining radiotherapy with immunotherapy. Front Mol Biosci. (2024) 11:1409300. doi: 10.3389/fmolb.2024.1409300 39044839 PMC11263218

[13] Hang ZS Huang YY Song A Sun ZJ . Radiotherapy elicits immunogenic cell death and metabolic shifts in the tumor microenvironment: implications for immunotherapy. Int J Med Sci. (2025) 22:3277–91. doi: 10.7150/ijms.109515 40765564 PMC12320787

[14] Vohra V Dhanawat M Chalotra R Bhushan B Garima Girdhar J . Shaping immunotherapy through the tumor microenvironment: Translational perspectives. Crit Rev Oncol Hematol. (2026) 220:105169. doi: 10.1016/j.critrevonc.2026.105169 41628747

[15] Antonia SJ Villegas A Daniel D Vicente D Murakami S Hui R . Durvalumab after chemoradiotherapy in stage III non-small-cell lung cancer. N Engl J Med. (2017) 377:1919–29. doi: 10.1056/NEJMoa1709937 28885881

[16] Faivre-Finn C Vicente D Kurata T Planchard D Paz-Ares L Vansteenkiste JF . Four-year survival with durvalumab after chemoradiotherapy in stage III NSCLC-an update from the PACIFIC trial. J Thorac Oncol. (2021) 16:860–7. doi: 10.1016/j.jtho.2020.12.015 33476803

[17] Yerolatsite M Torounidou N Amylidi AL Rapti IC Zarkavelis G Kampletsas E . A systematic review of pneumonitis following treatment with immune checkpoint inhibitors and radiotherapy. Biomedicines. (2025) 13:946. doi: 10.3390/biomedicines13040946 40299683 PMC12025308

[18] Guberina N Wirsdörfer F Stuschke M Jendrossek V . Combined radiation- and immune checkpoint-inhibitor-induced pneumonitis - The challenge to predict and detect overlapping immune-related adverse effects from evolving laboratory biomarkers and clinical imaging. Neoplasia. (2023) 39:100892. doi: 10.1016/j.neo.2023.100892 37011458 PMC10124136

[19] Jiang N Zhang J Guo Z Wu Y Zhao L Kong C . Short-course neoadjuvant radiotherapy combined with chemotherapy and toripalimab for locally advanced esophageal squamous cell carcinoma (SCALE-1): a single-arm phase Ib clinical trial. J Immunother Cancer. (2024) 12:e008229. doi: 10.1136/jitc-2023-008229 38199609 PMC10806572

[20] Janjigian YY Shitara K Moehler M Garrido M Salman P Shen L . First-line nivolumab plus chemotherapy versus chemotherapy alone for advanced gastric, gastro-oesophageal junction, and oesophageal adenocarcinoma (CheckMate 649): a randomised, open-label, phase 3 trial. Lancet. (2021) 398:27–40. doi: 10.1016/S0140-6736(21)00797-2 34102137 PMC8436782

[21] Meng X Sun H Cui T Huang W . Tislelizumab for consolidation therapy in patients with pathologically residual Esophageal Squamous Cell Carcinoma after definitive concurrent Chemoradiotherapy: a multicenter, randomized, controlled phase II trial. Future Oncol. (2024) 20:3397–402. doi: 10.1080/14796694.2024.2432290 39589855 PMC11776850

[22] Yang F Dan M Shi J Fan L Zhang H Jian T . Efficacy and safety of PD-1 inhibitors as second-line treatment for advanced squamous esophageal cancer: a systematic review and network meta-analysis with a focus on PD-L1 expression levels. Front Immunol. (2025) 15:1510145. doi: 10.3389/fimmu.2024.1510145 39916953 PMC11798917

[23] Shah MA Kennedy EB Deighton D Jhawer M Kadakia KC Mukherjee S . Immunotherapy and targeted therapy for advanced gastroesophageal cancer: ASCO guideline update. J Clin Oncol. (2026) 44(12):1145–65. doi: 10.1200/JCO-25-02958 41747202

[24] Lee J Kim B Jung HA La Choi Y Sun JM . Nivolumab for esophageal squamous cell carcinoma and the predictive role of PD-L1 or CD8 expression in its therapeutic effect. Cancer Immunol Immunother. (2021) 70:1203–11. doi: 10.1007/s00262-020-02766-7 33123755 PMC10992948

[25] Jo NC Shroff GS Ahuja J Agrawal R Price MC Wu CC . Radiation recall pneumonitis: imaging appearance and differential considerations. Korean J Radiol. (2024) 25:843–50. doi: 10.3348/kjr.2024.0334 39197829 PMC11361796

[26] Hu Q Wang S Ma L Sun Z Liu Z Deng S . Radiological assessment of immunotherapy effects and immune checkpoint-related pneumonitis for lung cancer. J Cell Mol Med. (2024) 28:e17895. doi: 10.1111/jcmm.17895 37525480 PMC10902575

[27] Abdul Hamid MF Che Rahim MJ Arif N Mohamad Jailaini MF . Sequential radiation-induced and immune checkpoint inhibitor-induced pneumonitis in a patient with breast carcinoma. BMJ Case Rep. (2026) 19:e269851. doi: 10.1136/bcr-2025-269851 41545225

[28] Chinese Thoracic Society Chinese Association for Cancer Rehabilitation & Respiratory Oncology Beijing Cancer Prevention and Treatment Society . Expert consensus on diagnosis and management of immune checkpoint inhibitor-related pneumonitis (2025). Chin J Tuberculosis Respir Dis. (2025) 48:329–43. doi: 10.3760/cma.j.cn112147-20241121-00694 40159051

[29] Chinese Society of Clinical Oncology Immunotherapy Committee Chinese Society of Digital Medicine Chongqing Association of Integrative Medicine for Tumor Immunotherapy . Expert consensus on diagnosis and management of interstitial pneumonia induced by thoracic radiotherapy combined with immune checkpoint inhibitors (2026 edition). Chin J Radiological Med Prot. (2026) 46:213–29. doi: 10.3760/cma.j.cn112271-20250813-0029530704229

[30] Hripcsak G Zhang L Chen Y Li K Suchard MA Ryan PB . Assessing covariate balance with small sample sizes. Stat Med. (2025) 44:e70212. doi: 10.1002/sim.70212 40772805 PMC13036809

[31] Stuart EA Lee BK Leacy FP . Prognostic score-based balance measures can be a useful diagnostic for propensity score methods in comparative effectiveness research. J Clin Epidemiol. (2013) 66:S84–S90.e1. doi: 10.1016/j.jclinepi.2013.01.013 23849158 PMC3713509

[32] Xie J Liu C . Adjusted Kaplan–Meier estimator and log-rank test with inverse probability of treatment weighting for survival data. Stat Med. (2005) 24:3089–110. doi: 10.1002/sim.2174 16189810

[33] Hou Y Yang K Wang L Wang J Huang X Piffkó A . Radiotherapy enhances metastasis through immune suppression by inducing PD-L1 and MDSC in distal sites. Clin Cancer Res. (2024) 30:1945–58. doi: 10.1158/1078-0432.CCR-23-3206 38427437 PMC11062826

[34] Guo S Yao Y Tang Y Xin Z Wu D Ni C . Radiation-induced tumor immune microenvironments and potential targets for combination therapy. Signal Transduct Target Ther. (2023) 8:205. doi: 10.1038/s41392-023-01462-z 37208386 PMC10199044

[35] Guo X Ma S Wang J Fu Y Ma W . Terminally exhausted CD8^+^ T cells in solid tumors: biology, biomarker potential and translational tools for precision oncology. Front Immunol. (2026) 16:1709852. doi: 10.3389/fimmu.2025.1709852 41601689 PMC12833282

[36] Cogels MM de Caluwé A Buisseret L Penninckx S . Unlocking the therapeutic potential of immuno-radiotherapy: insights from preclinical and clinical research. Oncoimmunology. (2026) 15:2608436. doi: 10.1080/2162402X.2025.2608436 41491954 PMC12778880

[37] Mannion J Meier P . Harnessing immunogenic cell death to boost the impact of radiation therapy. Cancer J. (2025) 31:e0780. doi: 10.1097/PPO.0000000000000780 40768307

[38] Shah MA Bennouna J Doi T Shen L Kato K Adenis A . KEYNOTE-975 study design: a Phase III study of definitive chemoradiotherapy plus pembrolizumab in patients with esophageal carcinoma. Future Oncol. (2021) 17:1143–53. doi: 10.2217/fon-2020-0969 33533655 PMC7927908

[39] Yu R Wang W Li T Li J Zhao K Wang W . RATIONALE 311: tislelizumab plus concurrent chemoradiotherapy for localized esophageal squamous cell carcinoma. Future Oncol. (2021) 17:4081–9. doi: 10.2217/fon-2021-0632 34269067

[40] Spigel DR Faivre-Finn C Gray JE Vicente D Planchard D Paz-Ares L . Five-year survival outcomes from the PACIFIC trial: durvalumab after chemoradiotherapy in stage III non-small-cell lung cancer. J Clin Oncol. (2022) 40:1301–11. doi: 10.1200/JCO.21.01308 35108059 PMC9015199

[41] Bradley JD Sugawara S Lee KH Ostoros G Demirkazik A Zemanova M . Simultaneous durvalumab and platinum-based chemoradiotherapy in unresectable stage III non-small cell lung cancer: the phase III PACIFIC-2 study. J Clin Oncol. (2025) 43:3610–21. doi: 10.1200/JCO-25-00036 41082707 PMC12622289

[42] Wang J Cheng YJ Wang JN Li J Wang W Wang X . Consolidative camrelizumab following definitive concurrent chemoradiotherapy with involved-field irradiation in locally advanced esophageal squamous cell carcinoma: a single-arm phase 2 trial. J Clin Oncol. (2025) 43:abstr 2597. doi: 10.1200/JCO.2025.43.16_suppl.2597 42031221

[43] Xu RH Cho BC Chen M Muro K Wyrwicz L Li B . Atezolizumab with or without tiragolumab in unresectable esophageal squamous-cell carcinoma following definitive concurrent chemoradiotherapy (SKYSCRAPER-07): a randomised, phase III study. Ann Oncol. (2026). S0923-7534(26)01431-6. doi: 10.1016/j.annonc.2026.07.413 42628839

[44] Yu N Chen C Wang X Ge X Yang X Li J . Impact of consolidation immunotherapy after definitive chemoradiotherapy in locally advanced esophageal squamous cell carcinoma: a real-world multicenter study. Int Immunopharmacol. (2025) 167:115666. doi: 10.1016/j.intimp.2025.115666 41086556

[45] Japan Esophageal Society . Japanese classification of esophageal cancer, 11th edition: part I. Esophagus. (2017) 14:1–36. doi: 10.1007/s10388-016-0551-7 28111535 PMC5222932

[46] Mine S Tanaka K Kawachi H Shirakawa Y Kitagawa Y Toh Y . Japanese classification of esophageal cancer, 12th edition: part I. Esophagus. (2024) 21:179–215. doi: 10.1007/s10388-024-01054-y 38568243 PMC11199297

[47] Mine S . Review of the Japanese classification of esophageal cancer 12th edition, and proposals for the 13th edition. Ann Thorac Cardiovasc Surg. (2024) 30:24–00061. doi: 10.5761/atcs.ra.24-00061 38945946 PMC11219218

[48] Takahashi N Okamura A Terayama M Kato T Ishida H Kanamori J . Diagnostic performance of computed tomography short-axis diameter for supraclavicular lymph node metastasis after neoadjuvant chemotherapy for esophageal squamous cell carcinoma. Dis Esophagus. (2025) 38:doaf121. doi: 10.1093/dote/doaf121 41396847

[49] Ding Y Jing W Zhu H . Radiotherapy-induced PD-L1 conversion in esophageal squamous cell carcinoma: From multidimensional mechanisms to clinical rescue strategies. Biochim Biophys Acta Rev Cancer. (2026) 1881(6):189703. doi: 10.1016/j.bbcan.2026.189703 42778075

[50] Guo X Wei Y Miao Z Ma S Ma W . Peripheral blood biomarkers in PD-1/PD-L1 immunotherapy: distinguishing predictive from prognostic biomarkers. Front Immunol. (2026) 17:1924506. doi: 10.3389/fimmu.2026.1924506 42724816 PMC13559439

[51] Chen G Huang AC Zhang W Zhang G Wu M Xu W . Exosomal PD-L1 contributes to immunosuppression and is associated with anti-PD-1 response. Nature. (2018) 560:382–6. doi: 10.1038/s41586-018-0392-8 30089911 PMC6095740

[52] Xu S Huang Z Li Y Lu L Ma W . Emerging biomarkers in melanoma: Bridging molecular discovery and precision oncology. Cancer Lett. (2026) 645:218359. doi: 10.1016/j.canlet.2026.218359 41734832

[53] Wang X He J Li J Wu C Yue M Niu S . Concordance of assessments of four PD-L1 immunohistochemical assays in esophageal squamous cell carcinoma (ESCC). J Cancer Res Clin Oncol. (2024) 150:43. doi: 10.1007/s00432-023-05595-0 38280970 PMC10821831

[54] Xue L Li Y Jiang L Liu C Cheng N Guo C . Concordance between four PD-L1 immunohistochemical assays and 22C3 pharmDx assay in esophageal squamous cell carcinoma in a multicenter study. J Natl Cancer Cent. (2023) 4:162–8. doi: 10.1016/j.jncc.2023.11.003 39282585 PMC11390698

[55] Chen L Wang Y Zheng Q Wang Y Jin Y Li Y . Consistency analysis of PD-L1 immunohistochemistry antibodies in esophageal squamous cell carcinoma. China Oncol. (2023) 33:469–77. doi: 10.19401/j.cnki.1007-3639.2023.05.007

[56] Li N Sohal D . Current state of the art: immunotherapy in esophageal cancer and gastroesophageal junction cancer. Cancer Immunol Immunother. (2023) 72:3939–52. doi: 10.1007/s00262-023-03566-5 37995002 PMC10991203

[57] Ciammella P Cozzi S Borghetti P Galaverni M Nardone V Ruggieri MP . Redetermination of PD-L1 expression after chemio-radiation in locally advanced PDL1 negative NSCLC patients: retrospective multicentric analysis. Front Oncol. (2024) 14:1325249. doi: 10.3389/fonc.2024.1325249 38357196 PMC10866304

