# Supplementary material for: Efficacy and safety analysis of consolidation immunotherapy following definitive concurrent chemoradiotherapy for locally advanced esophageal squamous cell carcinoma
Source: Front Immunol. 2026 Oct 19;17:1983549. doi: 10.3389/fimmu.2026.1983549 (PMC13724329; doi:10.3389/fimmu.2026.1983549)
Supplement: Supplementary file 1 [file DataSheet1.pdf]

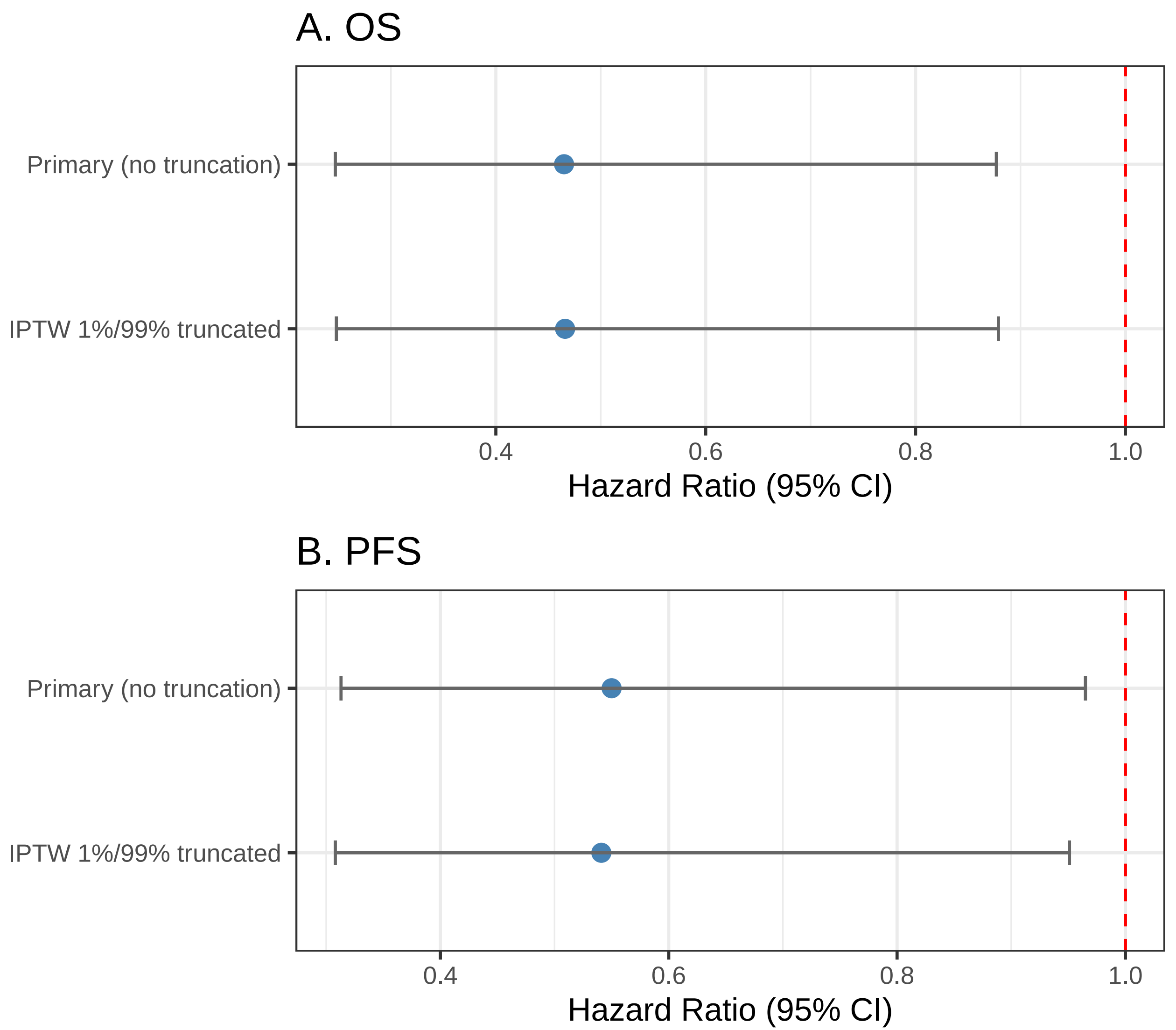

A. OS
Primary (no truncation)
IPTW 1%/99% truncated
0.4
0.6
0.8
1.0
Hazard Ratio (95% CI)
B. PFS
Primary (no truncation)
IPTW 1%/99% truncated
0.4
0.6
0.8
1.0
Hazard Ratio (95% CI)
