# Supplementary material for: Efficacy and safety analysis of consolidation immunotherapy following definitive concurrent chemoradiotherapy for locally advanced esophageal squamous cell carcinoma
Source: Front Immunol. 2026 Oct 19;17:1983549. doi: 10.3389/fimmu.2026.1983549 (PMC13724329; doi:10.3389/fimmu.2026.1983549)

M0 cohort: PFS and OS

A

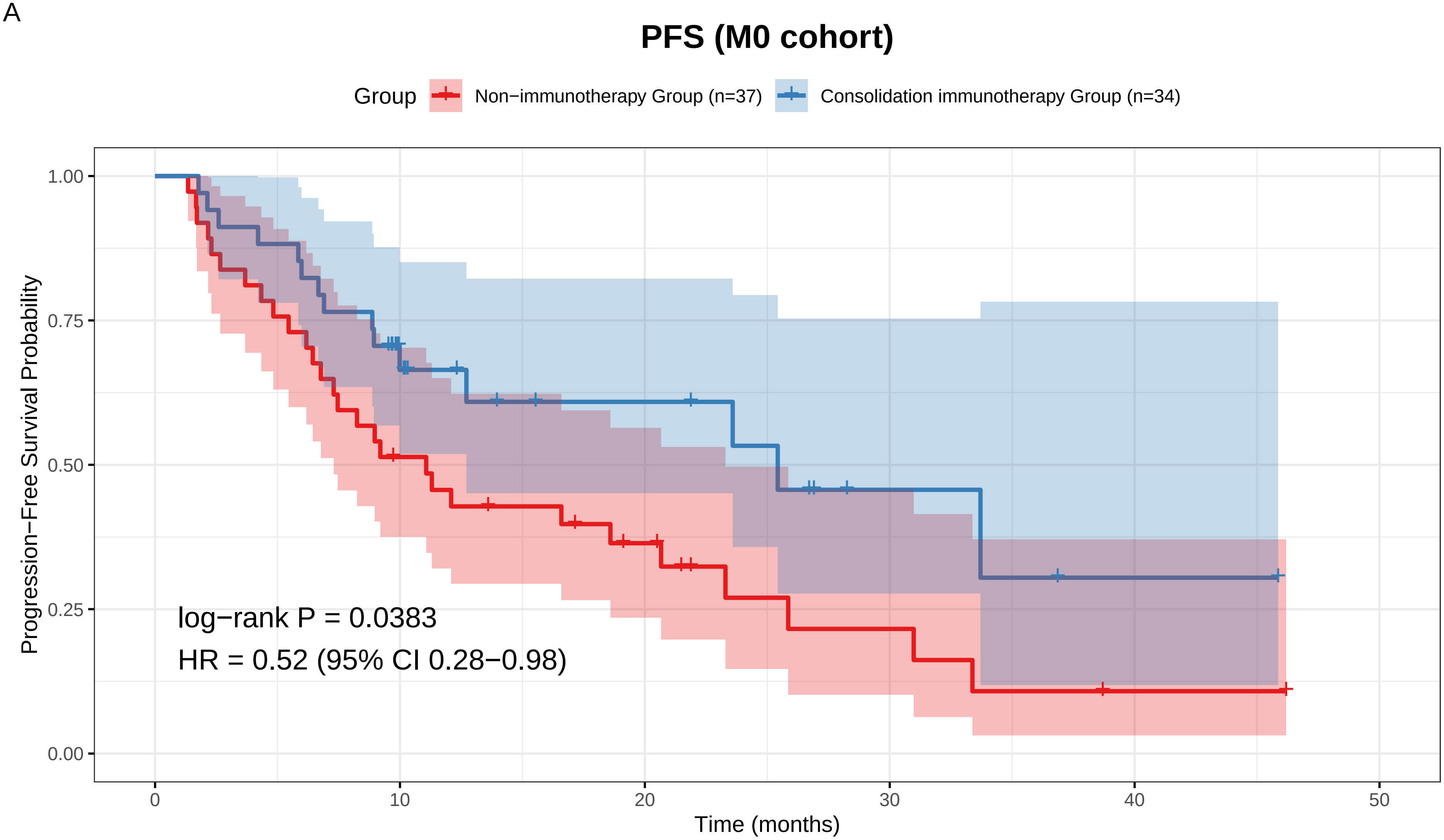

B

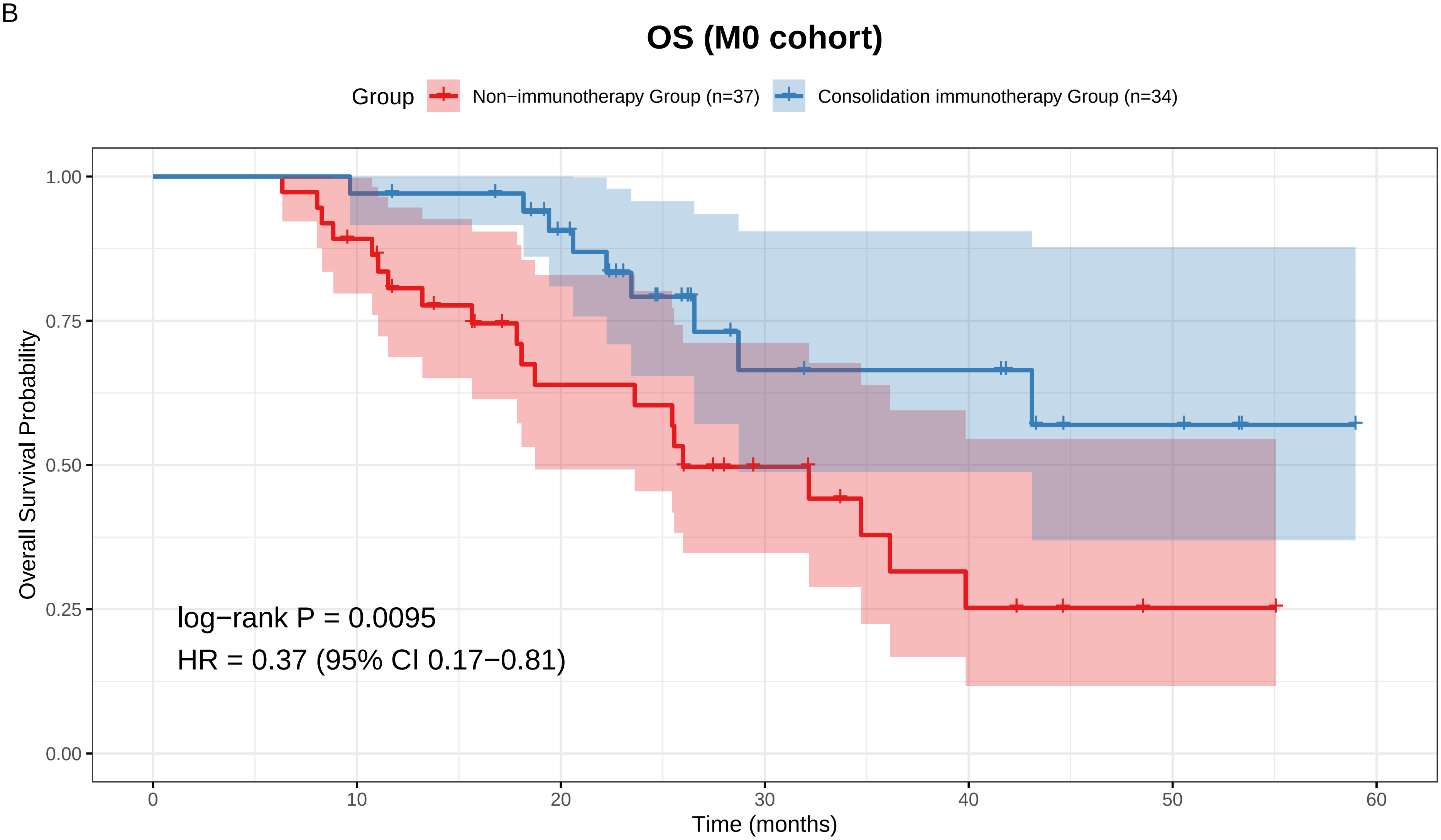

Supplement: Supplementary file 2 [file DataSheet2.pdf]
